# The Protein Extract of Chlorella minutissima Inhibits The Expression of *MMP-1, MMP-2* and *MMP-9* in Cancer Cells through Upregulation of *TIMP-3* and Down Regulation of *c-Jun*

**DOI:** 10.22074/cellj.2018.5277

**Published:** 2018-03-18

**Authors:** Mugdha Kunte, Krutika Desai

**Affiliations:** 1 Department of Biological Sciences, NMIMS University, Vile Parle (W), Mumbai, India; 2 Department of Microbiology, Mithibai College, Vile Parle (W), Mumbai, India

**Keywords:** Chlorella spp., Matrix Metalloproteinases, Microalgae, Tissue Inhibitor of Metalloproteinases, Transcription Factor AP-1

## Abstract

**Objective:**

Considering the bioactivities exhibited by microalgae, the effect of protein extract of Chlorella minutissimma (CP extract) was investigated on the expression of human matrix metalloproteinases-1 (*MMP-1*) in the breast cancer cell line MDA-MB231, and that of *MMP-2* and *-9* in hepatocellular cancer cell line HepG2 at different expression levels. The study aimed identification and analysis of inhibitory activity of microalgal components extracted from *Chlorella minutissima against* human MMPs.

**Materials and Methods:**

In this experimental study, we analysed the effect of *Chlorella* extracts on *MMP-1, -2,* and *-9* expression at various levels. Gelatin zymography was performed to study the inhibitory effect of Chlorella exracts on human gelatinases at the activity level, followed by western blotting to analyse the expression of all three MMPs at the protein level. The similar effect at the mRNA level along with the probable mechanism underlying inhibition of MMPs was assessed using real-time polymerase chain reaction (PCR).

**Results:**

The results reveal that the treatment with CP extract decreased the mRNA expression of *MMP-1, MMP-2,* and *MMP-9* by 0.26-, 0.29-, and 0.40-fold, respectively, at 20 μg/ml concentration as well as inhibited the activity of *MMP-2* and *MMP-9 *by 37.56 and 42.64%, respectively, at 15 μg/ml concentration. Additionally, upregulated mRNA expression of tissue inhibitor of metalloproteinases-3 (*TIMP-3*) by 1.68-fold was seen in HepG2 cells at 20 μg/ml concentration treatment group. However, CP extract did not induce any change in the mRNA expression of the *TIMP-1, -2* and *-4* in HepG2 and *TIMP-1, -2, -3* and *-4* in MDA-MB231 cells. Activator protein-1 (AP-1)-dependent c-Jun-mediated transcriptional regulation of *MMP-1, -2,* and *-9* was also studied to elucidate the appropriate mechanism involved in the inhibition of MMPs.

**Conclusion:**

The CP extract successfully inhibited *MMP-1, -2*, and *-9* at different expression levels through TIMP-3 upregulation and *c-Jun* downregulation.

## Introduction

Matrix metalloproteinases (MMPs) are the essential matrix proteases involved in migration and proliferation of cells by degradation of matrix components. The tightly regulated expression of MMPs is essential for organ development and tissue remodelling. Besides their positive roles in the normal physiology of cells, they have been identified as significant prognostic markers in diseases, such as cancer (1, 2). Hence, MMPs were identified as potential targets to control invasive and metastatic cancers (3, 4). Regulation of MMPs in pathogenic conditions using MMP inhibitors (MMPIs) derived from synthetic as well as a natural sources (5, 6) has been widely studied previously.

shifted from the use of algae a superfoods to that as fuel- producing bio-resources. Besides their notable role in the bio-fuel production industry, their biological activities in medicine and pharmaceutics also have received increasing attention in recent years. *Chlorella* species were explored for various activities such as anti-inflammatory, antiangiogenic anti-proliferative, anti-ageing, anti-oxidant, apoptosis inducing activities (7-13). 

*Chlorella* extracts were studied previously for their inhibitory activity on *MMP-1* expression induced by ultra violet ß rays (UVB) exposure in skin fibroblasts (7). However, *Chlorella* extract has not been studied previously for its effect on MMPs from other classes. Hence, the current study aimed examination of inhibitory activity of protein extracts of *Chlorella minutissima* (CP extracts) on human *MMP-2* and *MMP-9* at mRNA, protein, as well as activity level in HepG2 cells, and that of *MMP-1* at protein and mRNA expression levels in MDA-MB231 cells. 

Additionally, the inhibition of gelatinase activity was studied with respect to change in expression of endogenous MMPIs (*TIMP-1, -2, -3,* and *-4*) in HepG2 cells. Additionally, the activator protein-1 (AP-1)dependent transcriptional regulation of* MMP-1, -2,* and *-9 *was investigated using real-time polymerase chain reaction (PCR) analysis.

## Materials and Methods

### Maintenance of microalgae 

In this experimental study, *C. minutissima* strain was obtained from Indian Agricultural Research Institute (IARI), New Delhi, India. The strain was cultured in BG-11 medium at 28°C in continuous light with 8 kLux intensity and maintained under similar conditions on BG-11 agar. The growing culture was harvested every alternate day to monitor its growth using parameters, such as wet cell biomass and protein content, for 15 days.

### Preparation of Chlorella protein extract 

*C. minutissima* was cultured in BG-11 medium for 15 days and then the wetbiomass was harvested and used for the further studies. The wet biomass obtained was soaked in MilliQ water and heated at a constant temperature of 60°C for 30 minutes. Further, it was incubated in 0.1 N NaOH for 15 minutes under similar temperature conditions. After alkali treatment cell debris were removed by centrifugation at 5000 ×g at 4°C and the cell free extract of proteins was used for further studies. The protein extract was fractionated using ammonium sulphate precipitation and the proteins were isolated into different fractions, such as 0-30%, 30-60%, and 60-90%. These precipitates were dialysed at 4°C using phosphate buffered saline pH=8. These dialysed fractions were evaluated using gelatin zymography for their activity against MMP-2 and *-9* (data not shown). 

The results revealed that the activity was retained in the crude protein extract; however, the isolated fractions lost the activity. Therefore, the crude protein extract was selected for further experiments in the current study. The extract obtained was filtered through 0.22-µm membrane filter, freeze dried and stored at -20°C until further use. The total protein concentration of the cell free extract (CP extract) was determined using Lowry’s method.

### Maintenance and culturing of human cell lines

The MDA-MB 231 and HepG2 cells were obtained from (authenticated and maintained by) National Centre for Cell Science (NCCS), Pune, India and was cultured in Dulbecco’s modified Eagle’s medium (DMEM, Sigma-Aldrich, USA) supplemented with heat-inactivated fetal bovine serum (FBS, 10%, Genetix, India) and antibiotic solution of penicillin streptomycin (Sigma-Aldrich, USA). Trypsin-EDTA (Gibco, India) was used for trypsinisation of cells. All materials used were of cell culture grade.

### Cytotoxicity assay 

The cytotoxicity of CP extract was estimated using the methyl thiazoltetrazolium (MTT) assay. MDA-MB231 cells (7×10^3^/0.1 ml) and HepG2 cells (3×10^3^/0.1 ml) were seeded in each well of a 96-well plate and exposed to different concentrations (5-40 µg/0.1 ml) of CP extract. The untreated cells were maintained as control. After 24 hours of incubation at 37ºC, the cells were exposed to 20 µl of MTT (2 mg/ml) for 3 hours. Supernatant from each well was discarded and dimethyl sulphoxide (DMSO, 100 µl/well) was added to dissolve formazan crystals formed during the incubation step. The absorbance of all treated and untreated samples was measured using a microplate reader (Bio-Rad) at 570 nm. The data is represented as mean ± SD of three independent experiments in terms of percentage cellular viability of cells as compared to that of untreated controls.

### Non reducing gelatin zymography

Gelatin zymography was performed to analyse the gelatinolytic activity of metalloproteinases (*MMP-2* and *-9*) in HepG2 cells. The cells (1×10^6^/well) seeded in 6-well plates were exposed to different concentrations of CP extract (10, 15, 20, and 25 µg/ml) in serum free medium for 24 hours. The untreated cells were maintained as control. After incubation, cell-free supernatants were collected, aliquoted, and lyophilized, till further use. The samples were reconstituted in loading buffer and loaded on 10% sodium dodecyl sulfate (SDS)-polyacrylamide gel containing 1% gelatin. The MMPs resolved during electrophoresis were incubated in 25% Triton-X100 for 1hour, followed by incubation in substrate buffer [50 mM Tris-Cl (pH=7.6), 5 mM CaCl_2_] at 37°C overnight followed by staining and destaining for visual observation of a clear band against the blue-colored background along with respective protein markers. The band intensities were quantified with Image J software 5.0. 

### Western blotting

The expression of *MMP-1* (in MDA-MB 231 cells), and *MMP-2* and *-9* (in HepG2 cells) at the protein level was determined by western blotting. MDA-MB231 and HepG2 cells (1×10^6^/well) were cultured in 6 well plates and exposed to CPextract (10, 15, 20 and 25 µg/ml) in FBS free medium. The untreated cell control was maintained. The monolayer of cells was removed using mechanical scraping and incubated in lysis buffer, at 4°C. Protein samples (20 µg) obtained were loaded on 10% reducing and denaturing polyacrylamide gel for separation. The proteins resolved on the gel were transferred to nitrocellulose membrane (Amersham Biosciences, UK) by electroblotting at 18 mA for 16 hours at 4°C. 

The membrane was blocked with 5% BSA to avoid nonspecific binding followed by probing with rabbit monoclonal MMP-1, -2 and -9 antibodies (1:1000, Abcam, UK) on respective blots. After subsequent washing the blots were later probed with (1:2000, Merck, India). Goat anti-rabbit HRP-conjugated polyclonal secondary antibody. The chemiluminescence immerged through enzyme conjugated antibodies was captured on X-ray films. The mouse monoclonal ß-actin antibody (Sigma- Aldrich, USA) was maintained as an internal and loading control. The band intensities were quantified with Image J software 5.0.

### Real time polymerase chain reaction 

The expressions of* MMP-1* (inMDA-MB231 cells), and *MMP-2* and *-9* (in HepG2 cells) at the mRNA level were analysed using real-time PCR. MDA-MB231 and HepG2 cells (1×10^6^/well) were cultured in a 6-well plates and exposed to different concentrations of CP extract (15 and 20 µg/ml) in FBS-free medium. The untreated cell control was maintained during the experiment. After incubation total RNA was extracted using TRI-reagent (Sigma-Aldrich) and followed with complementary DNA synthesis using TAKARA, India cDNA synthesis kit. The expression of MMP (*MMP-1, -2,* and *-9*), TIMPs (*TIMP-1, -2, -3,* and *-4*), and transcription factor AP-1 (c-Jun and c-Fos) at the mRNA level before and after the treatment was assessed using real time PCR on Applied Biosystems Step One Plus thermocycler.

The primer sequences used for amplification of genes were:

MMP-1

F: 5´GAGCAAACACACTGACCTACAGGA3´ 

R: 5´TTGTCCCGATGATCTCCCCTGACA3´

MMP-2

F: 5´CAAGGACCGGTTTATTTGGC3´ 

 R: 5´ATTCCCTGCGAAGAACACAGC3´ 

MMP-9

F: 5´TGGGCTACGTGACCTATGAC3´ 

R: 5´CAAAGGTGAGAAGAGAGGGC3´ 

TIMP-1

F: 5´ACTTCCACAGGTCCCACAAC3´ 

R: 5´CACTGTGCATTCCTCACAGC3´ 

TIMP-2

F: 5´ATGCACATCACCCTCTGTGA3´ 

R: 5´CTCTGTGACCCAGTCCATCC3´ 

TIMP-3

F: 5´ACCGAGGCTTCACCAAGATG3´ 

R: 5´CATCATAGACGCGACCTGTCA3´

TIMP-4

F: 5´TACCAGGCTCAGCATTAT3´ 

 R: 5´CCACTTGGCACTTCTTATT3´ 

c-Jun-

F: 5´GGATCAAGGCGGAGAGGAAG3´

R: 5´GCGTTAGCATGAGTTGGAAC3´

c-Fos-

F: 5´GGGGCAAGGTGGAACAGTTA3´

R: 5´TTGGTCTGTCTCCGCTTGGA3´ 

The *GAPDH* with a primer sequence of

F: 5´CTCTCTGCTCCTCCTGTTCG 3´ 

R: 5´ACGACCAAATCCGTTGACTC3´ 

was used as an endogenous internal control.

The expression level of MMPs, TIMPs, c-Jun and c-Fos genes was normalised with *GAPDH*. An appropriate set of primers mentioned above were used to amplify respective genes using the following cycling conditions: 94°C for 5 minutes; followed by 35 cycles at 94°C for 30 seconds, 60°C for 30 seconds (for *MMP-1, -2, -9, TIMP-1, -2,* and *c-Jun *and *c-Fos*) and 58°C for 30 seconds (for *TIMP-3, -4*), and 72°C for 30 seconds extension. Melting curve was determined for the samples to verify change in products as per its specific melting temperature (T_m_). The data is represented in the form of fold change calculated using 2^-ΔΔCt^ method in treated samples with respect to that in the untreated control. All sets of reactions were performed in triplicates.

### Statistical analysis 

All the experiments were performed in triplicates and results are expressed in terms of mean ± SD. The data were analysed statistically using one-way ANOVAand Dunnett’spost test on GraphPad Prism software, (GraphPad software, Inc), P<0.05 was considered significant.

## Results

### Extraction and identification of Chlorella protein extract

The hot water extraction method was used to extract and isolate proteins from the wet cell biomass of *C. minutissima.* The total protein content of the CP extract was determined to be 5.086 mg/g of wet cell biomass. 

### Effect of Chlorella protein extract on cancer cell viability 

**Fig.1:**
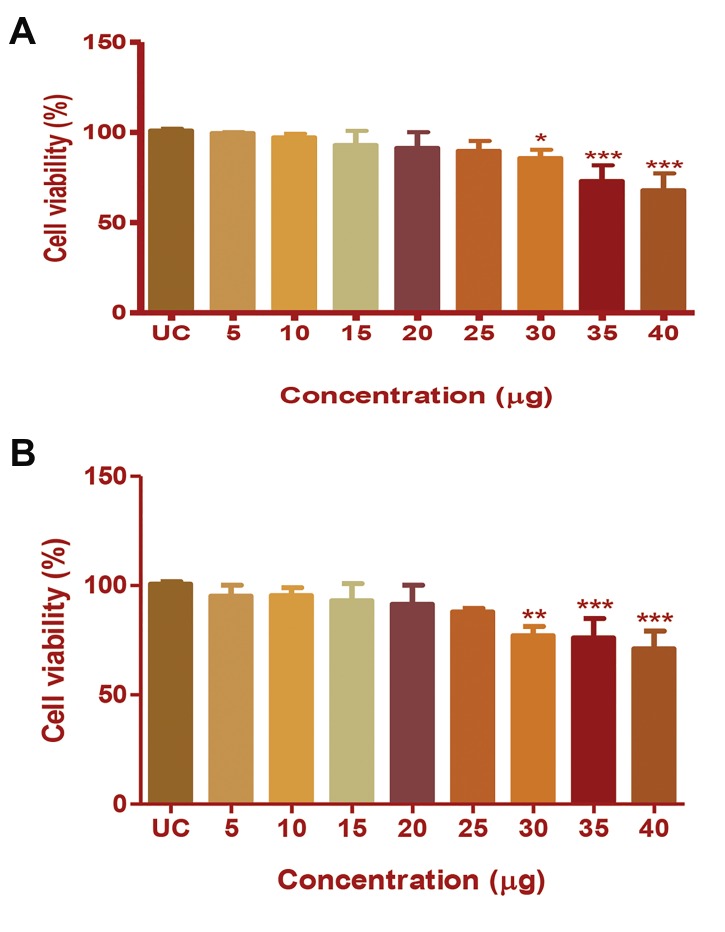
Cytotoxicity induced by Chlorella protein (CP) extract in HepG2 and MDA-MB231 cells. The percentage of cell viability of A. HepG2 cells and B. MDA-MB231 cells after CP extract treatment at different concentrations. The results were analysed by one-way ANOVA and Dunnett’spost test. *; P<0.05, **; P<0.01, and ***; P<0.001.

Cell viability was determined by MTT assay. The cytotoxic concentrations of the CP extract in HepG2 and MDA-MB231 cells were assessed by this assay, and the observed MMP inhibition was confirmed to not due to cellular toxicity. The results are represented in terms of percentage cell viability. Treatment with CP extract did not induce a significant change in the viability of HepG2 or MDA-MB231 cells in the concentration range of 5-25 µg/100 µl. At 35 µg/100 µl concentration, the viability of HepG2 cells was significantly reduced to 85% (P<0.001, Fig.1A), and at 30 µg/100 µl concentration, viability of MDA-MB231 cells decreased to 77% (P<0.001, Fig.1B). Therefore, the concentrations between 10-25 µg/100 µl were selected for further study and precisely lower concentration was avoided to reduce technical errors. 

### *Chlorella* protein extract treatment reduced *MMP-9, -2,* and *-1* expression at the protein level 

**Fig.2:**
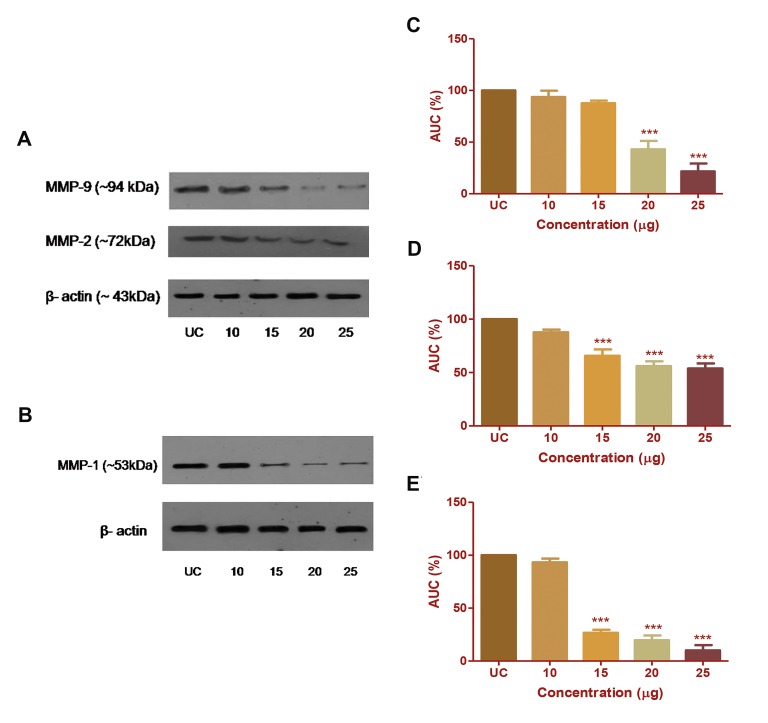
Effect of Chlorella protein (CP) extract treatment on the expression of *MMP-9* and *-2* in HepG2 cells and MMP-1 in MDA-MB 231 cells at the protein level. The effect of CP extract on A. MMP-9, MMP-2 in HepG2 cells and on B. MMP-1 in MDA-MB231 cells was observed by western blotting. The densitometric evaluation of C. MMP-9, D. MMP-2, and E. MMP-1 in terms of percentage band intensities depicted as mean ± SD of three individual experiments. The results were analysed by one-way ANOVA and Dunnett’spost test. ***; P<0.001, UC; Untreated control, and AUC; Area under curve.

All treated samples were analysed and expressed as percentage inhibition of MMP expression at the protein level in respective cancer cells, based on the intensity of the protein band. The band intensity of *MMP-1* and *MMP9* were significantly (P<0.001) inhibited by 69.89 and 51.82% at 20 µg/ml concentration of CP extract. At 15 µg concentration of CP extract, band intensities of *MMP-2* was inhibited by 45.98% (Fig.2A-E). No further change was observed in band intensities of all three MMPs at the protein level. The data obtained from three individual experiments indicates that the expression of *MMP-1, -2,* and *-9* was successfully reduced at the protein level after CP extract treatment. 

### *Chlorella* protein extract treatment inhibits enzymatic activity of *MMP-9* and *-2 *

**Fig.3:**
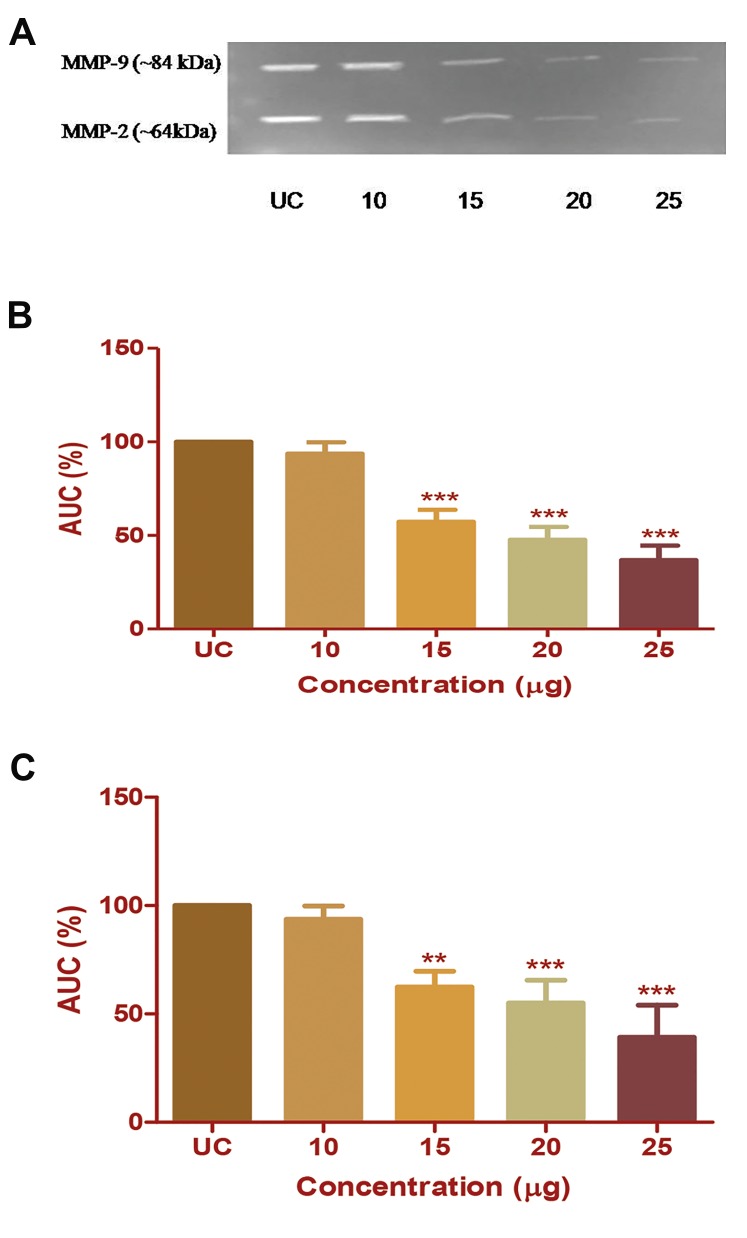
Effect of Chlorella protein (CP) extract on the gelatinolytic activity of *MMP-9* and *-2* in HepG2 cells. A. Band of clearance in gelatin zymogram. Densitometric analysis of the gelatinolytic activity of B. *MMP-9*, and C. *MMP-2*, respectively, with respect to untreated control (UC). The results were analysed by one-way ANOVA and Dunnett’spost test. **; P<0.01, ***; P<0.001, and AUC; Area under curve.

CP extract treatment causes inhibition of enzymatic activity of *MMP-2* and *-9* in HepG2 cells. The inhibition of enzymatic activity is expressed in terms of percentage inhibition of band intensities for *MMP-2* and *-9*. CP extract at a concentration of 15 µg/ml induced inhibition of *MMP-2* (~64 kDa) by 37.52% (P<0.001) and *MMP-9* (~84 kDa) by 42.13% (P<0.001). CP extract at 20 µg/ml concentration inhibits MMP-2 activity by 45% (P<0.001) and MMP-9 activity by 52% (P<0.001). No further change was observed in the percentage of band intensities with respect to *MMP-9* with increase in CP extract concentration. While, *MMP-2* was further inhibited up to 60% at CP extract concentration of 25 µg/ml (Fig.3A-C). The results obtained from three independent experiments have confirmed that CP extract successfully inhibits enzymatic activity of both the gelatinases in HepG2 cells without inducing cytotoxicity. 

### Chlorella protein extract treatment downregulates mRNA expression of MMP-1, -2, and *-9*

**Fig.4:**
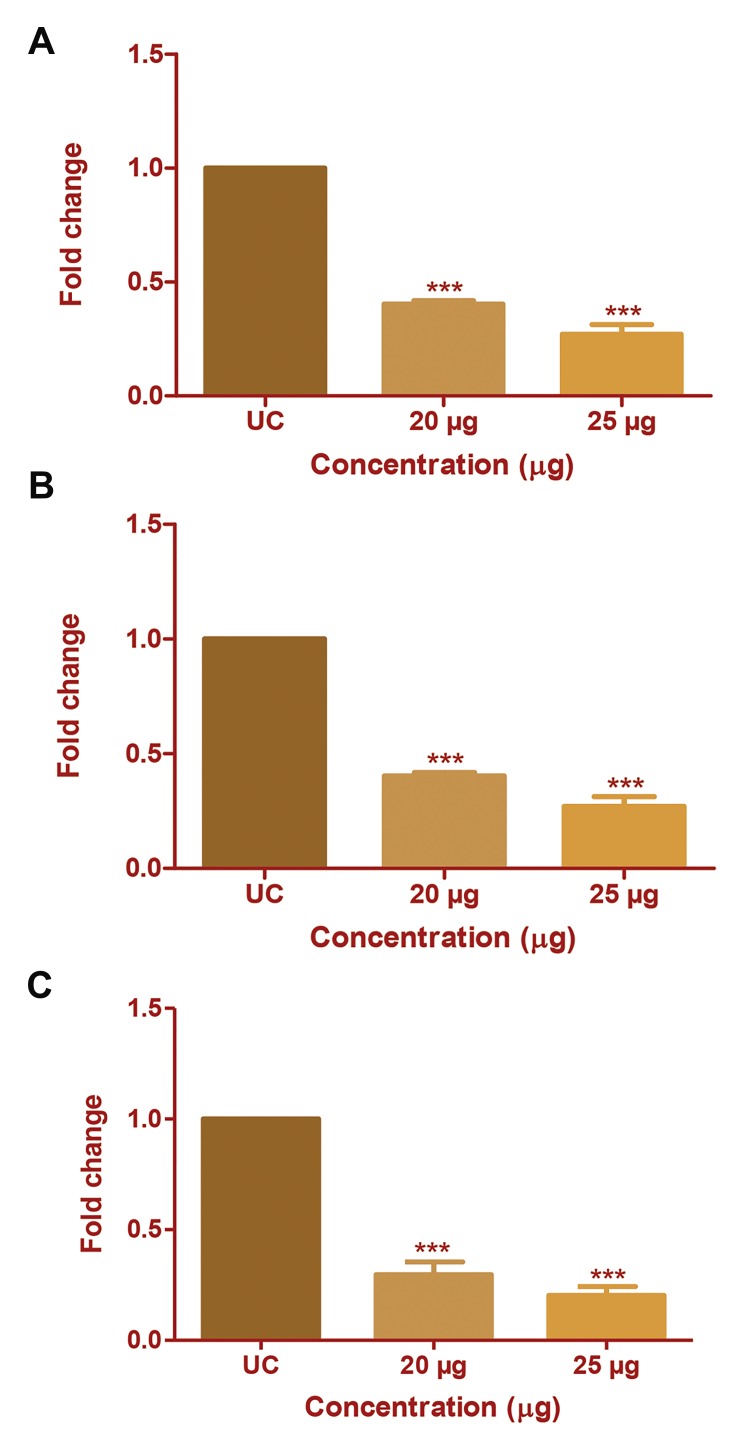
Effect of *Chlorella protein (CP)* extract on mRNA expression of *MMP- 1, -2* and *-9*. The change in mRNA expression of MMP-2 and *-9* after CP extract treatment was studied using real-time polymerase chain reaction (PCR). Fold change in mRNA expression of A. MMP-9 in HepG2 cells, B. *MMP-2* in HepG2 cells, and C. *MMP-1* in MDA-MB231 cells with respect to untreated control (UC). The results were analysed by one-way ANOVA and Dunnett’spost test. ***; P<0.001.

The effect of CP extract on mRNA expression of *MMP-1, MMP-2* and *MMP-9* was studied using real time PCR. Theanalysis was done at 20 and 25 µg/ml concentrations of CPextract which gave promising results in gelatin zymographyand western blotting. The CP extract significantly reduced, *MMP-9* expression by 0.49- and 0.27-fold (Fig.4A) MMP2 by 0.29- and 0.20-fold (Fig.4B) and MMP-1 by 0.26and 0.24-fold (Fig.4C) at 20 and 25 µg/ml concentrations, respectively. The study has confirmed that CP extract inhibits all three MMPs at the mRNA level of expression. 

### *Chlorella* protein extract treatment upregulates *TIMP-3* expression in HepG2 cells 

**Fig.5:**
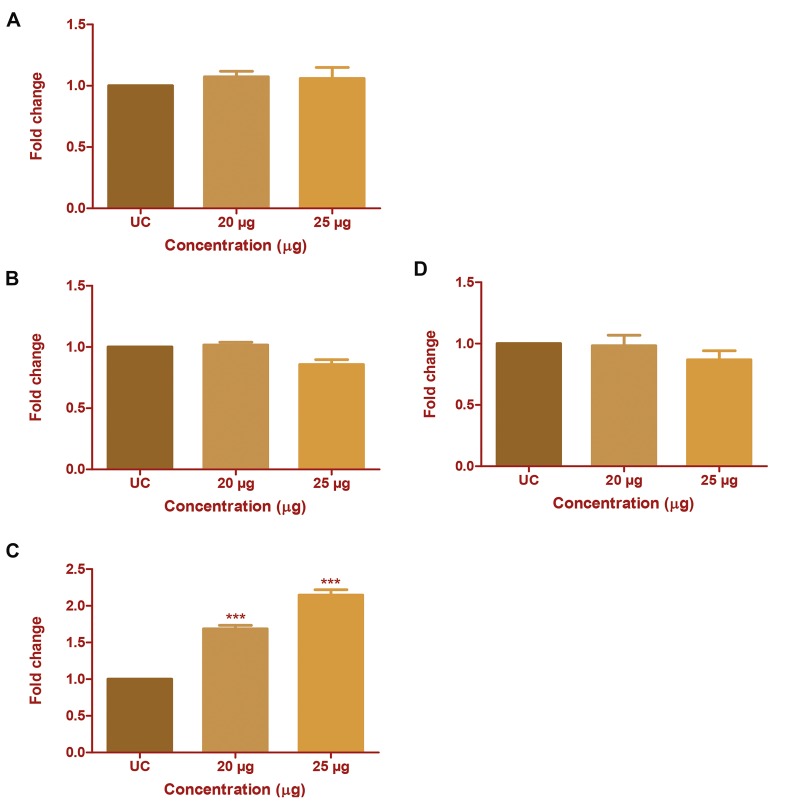
Effect of Chlorella protein (CP) extract on mRNA expression of *TIMP1, -2, -3*, and *-4*. The change in mRNA expression of TIMPs after CP extract treatment was studied using real-time polymerase chain reaction (PCR). Fold change in mRNA expression of A. TIMP-1, B. *TIMP-2,* C. *TIMP-3,* and D. *TIMP-4* with respect to untreated control (UC). The results were analysed by one-way ANOVA and Dunnett’s post test. ***; P<0.001.

The effect of CP extract on mRNA transcription from all the four TIMPs was studied using real-time PCR. Expression of TIMPs and that of MMPs is negatively correlated in cancer cells. Here, we investigated the change in transcription of TIMPs in HepG2 cells. CP extract treatment affected *TIMP-3* expression, although the expression of *TIMP-1, -2* and *-4* remained unaffected. Similarly, no change in expression of *TIMPs* was seen in MDA-MB231 cells (Fig. 5A-D). TIMP-3 expression was upregulated by 1.68-fold and 2.44-fold at 20 and 25 µg/ ml concentration of CP extract respectively. This suggests that TIMP-3 upregulation may have a positive impact on downregulation of *MMP-2* and *-9* at the enzyme activity level in HepG2 cells. 

### Effect of *Chlorella* protein extract on *c-Jun* and *c-Fos* expression in HepG2 and MDA-MB231 cells 

**Fig.6:**
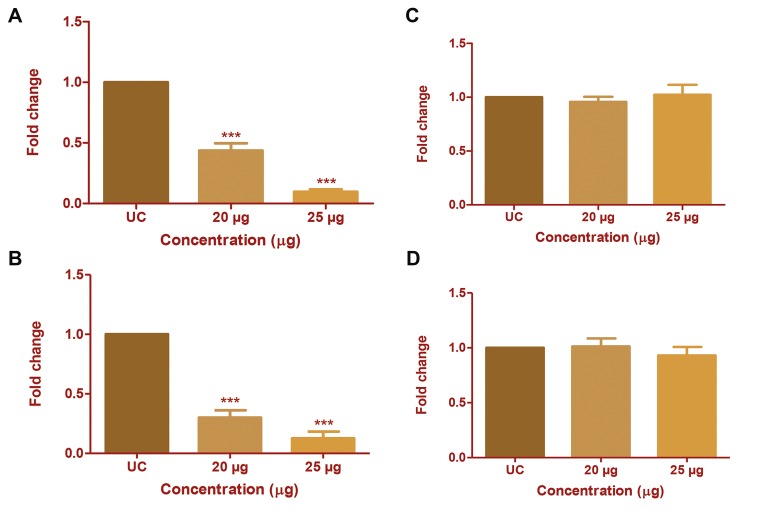
Effect of Chlorella protein (CP) extract on mRNA expression of *c-Jun* and *c-Fos*. The change in mRNA expression of *c-Jun* and *c-Fos* after CP extract treatment was studied using real-time polymerase chain reaction (PCR). Fold change in mRNA expression of *c-Jun* in A. HepG2 cells, B. MDAMB231 and *c-Fos* in, C. HepG2 cells, and D. MDA-MB231 with respect to untreated control (UC). The results were analysed by one-way ANOVA and Dunnett’spost test. ***; P<0.001.

The inhibitory effect of CP extract on mRNA transcriptionof the homodimers of the AP-1 trasncription factor (c-Jun and *c-Fos*) was assessed by real-time PCR. Treatment with 20 µg/ ml of CP extract significantly (P<0.001) reduced the mRNAtranscription of c-Jun by 0.43-fold in HepG2 cells and 0.3fold in MDA-MB231 cells, and the expression decreasedfurther with increase in CP extract concentration (Fig.6A, B). However, the mRNA transcription of *c-Fos* was unchanged following a similar treatment (Fig.6C, D). 

## Discussion

MMPs are present in several species ranging from viruses and bacteria to humans along with a conserved sequence at the active site motif. They are zinc binding proteases having structural similarities within the class. The MMPs discovered in humans until now are classified into sub-classes depending on their substrate specificity and structural arrangement. The class of gelatinases contains *MMP-2* and *MMP-9. MMP-1* falls into a group of collagenases possessing a capacity to cleave all types of fibrillar collagen proteins, such as type I, II, III, VII,VIII, and X. Structurally, collagenases have all the same subunits of gelatinases, except the fibronectin domain (4). 

Several studies have revealed that the expression of *MMP-1, -2,* and *-9* in cancers is regarded as a remarkable prognostic marker because activated MMP enzymes are almost undetectable under normal physiological conditions. *MMP-2* and *-9* are potential targets linked to migration and invasion of tumor cells (14-19). Similarly, over expression of MMP-1 in breast cancer (20, 21) is linked to their metastatic and invasive potential. These studies reported the unregulation of *MMP-2* and *MMP-9* in the HepG2 cell line and *MMP-1* in the MDA-MB231 cell lines. This makes these cell lines an important model to investigate the expression of *MMP-2, -9* and *-1* respectively. 

MMP expression should be modulated to avoid uncontrolled degradation of matrix components and to minimize the severity of cancer. The regulation of MMPs has been studied before using various MMPIs (22). Several of them failed due to the lack of a “targeted approach” towards unregulated MMPs under pathogenic conditions. In the last few years, MMPI studies have improved with newer insights (23, 24). Considering this background, in the current study, we attempted to analyse the inhibitory potential of a natural component extract against human MMPs at different levels of regulation, and to analyse the specificity of the extracts towards MMPs from two distinct classes. 

The enzymatic activity of MMPs is controlled by naturally expressed endogenous TIMPs. As compared to other compatible inhibitors found in other mammals, TIMPs found in the human genome are the most extensively studied class of proteins. TIMPs not only target MMPs, but also have a disintegrin and metalloproteinase with thrombospondin domains (ADAMs) as their non-MMP targets. Broadly, TIMPs are distributed as *TIMP-1, -2, -3,* and *-4* according to their sequence simililarity, affinity for protein targets, and dynamic function in cell signaling cascades, excluding the inhibitory roles (25). Induction of TIMP expression in cancer cells to overcome MMP over expression has been widely studied in the past (26, 27). 

In the current study, we investigated *MMP-2* and *-9* inhibition in their active forms with respect to the induction of mRNA transcription of TIMPs due to CP extract treatment. *TIMP* expression is negatively correlated to MMP expression in cancer cells. Here, we investigated the change in transcriptional expression of TIMPs in HepG2 and MDA-MB231 cells. Due to CP extract treatment TIMP-3 expression was observed to be affected only in HepG2 cells, although the expression of *TIMP-1, -2*, and *-4* was changed in both the cell lines. Interestingly,* TIMP-3* expression in HepG2 cells was dramatically upregulated in accordance with the increase in CP extract concentration. This suggests that there may be a positive impact from *TIMP-3* upregulation on and *MMP-2* and *-9* downregulation, at the enzyme activity level, in HepG2 cells. 

According to previous studies, TIMP-3 is a broad spectrum inhibitor of MMPs. Mino et al. have reported that inibition of mRNA expression of MMP-2 and *-9* is linked to upregulation of TIMP-3 expression (28). It has been reported that TIMP-3 suppresses angiogenesis in lung cancer by downregulating *MMP-2* (29). A study conducted by Neill et al. (30) reported that induction of TIMP-3 due to decorin (proteoglycan) certainly inhibits pro-angiogenic proteases, *MMP-2* and *-9*, in MDA-MB231 breast cancer cell lines. Several sources of evidence suggest that the induction of *TIMP-3* may inhibit *MMP-2* and *MMP-9* activity in cancer cells. Hence, collectively, we can conclude that the dramatic increase in *TIMP-3* expression in HepG2 cells might be one of the causes for inhibition of the activity of *MMP-2* and *MMP-9*.

Activator protein-1 (AP-1) is a known positive regulator of MMPs. It has been indicated that, AP-1 regulates basal as well as transactivated (through external stimuli, such as phorbolmyristate acetate, cytolines, and growth factors) expression of MMPs (31). Several biological components have been studied previously for trascriptional regulation of MMPs through AP-1-activated pathways.

Chrysin, a chemical extracted from a plant source inhibited *MMP-9* expression through inhibition of the AP-1 trasncription factor in gastric cancer cells (32). Berberine, a Chinese medicinal herb, inhibited migration of human smooth muscle cells by reducing mRNA transcription of *MMP-2* and *-9* by interrupting AP-1 and NF-.B signalling pathways (33). Accordingly, in the current study, we have investigated the change in expression of the AP-1 transcription factor due to CP extract treatment. AP-1 is a heterodimer consisting of proteins from the *c-Jun* and *c-Fos* families. MMP transcription can be regulated through activation, phosphorylation, and translocation of *c-Jun* and *c-Fos* family members. Additionally, *c-Jun* activation is regulated by *c-Jun* N-terminal kinases (JNKs)mediated pathway (34), and the extracellular signal- regulated kinases (ERKs) phosphorylate and activate the c-Fos protein family (35).

Based on this background information, we investigated the change in expression of the members belonging to the AP-1 family. The CP extract treatment down regulated mRNA transcription of the *c-Jun* homodimer. However, c-Fos expression was not affected in either of the cell lines. This may indicate that *MMP-1, -2,* and *-9* were suppressed at the gene level by down regulation of *c-Jun* through a JNK-mediated pathway, although other members of this pathway should also be studied to understand the complete mechanism of action of CP extract.

## Conclusion

Collectively, the results of this study confirmed that CP extract successfully inhibits *MMP-1, -2,* and *-9* expression at the mRNA as well as the protein level and inhibits *MMP-2* and *-9* in their active forms. This indicates that CP extract is not selective for gelatinases or MMP-1 in terms of its inhibitory activity, although further studies are required to conclude its inhibitory activity towards MMPs from a distinct class. CP extract triggered MMP inhibition without inducing any cytotoxic effect. The current study also states that the activity of *MMP-2* and *MMP-9* may have been inhibited due to TIMP-3 upregulation. Additionally, CP extract treatment also suppressed mRNA transcription of *c-Jun*, in turn suppressing* MMP-1, -2,* and *-9* at the gene level, although the complete underlying mechanism is still to be investigated in the future. The current research would form a basis for conducting further studies on the identification and isolation of specific MMPIs from a natural source, which will help researchers achieve milestones in this field.
